# Multiple reasons underlaying uncontrolled disease in the majority of chronic rhinosinusitis patients

**DOI:** 10.3389/falgy.2022.1048385

**Published:** 2022-12-13

**Authors:** An-Sofie Viskens, Tine Wils, Pauline Van Bulck, Leen Cools, Olivier Vanderveken, Peter W. Hellings

**Affiliations:** ^1^ Laboratory of Allergy and Clinical Immunology Research Unit, Department of Microbiology, Immunology and Transplantation, KU Leuven, Leuven, Belgium; ^2^ Faculty of Medicine and Health Sciences, University of Antwerp, Antwerp, Belgium; ^3^ Department of Otorhinolaryngology, Head and Neck Surgery, UZ Leuven, Leuven, Belgium; ^4^ Department of Ear-Nose-Throat, Head and Neck Surgery, Antwerp University Hospital, Edegem, Belgium; ^5^ Department of Otolaryngology, Academic Medical Center, University of Amsterdam, Amsterdam, Netherlands; ^6^ Upper Airways Research Laboratory, Department of Otorhinolaryngology, Faculty of Medicine and Health Sciences, Ghent University, Ghent, Belgium

**Keywords:** chronic rhinosinusitis, uncontrolled disease, SCUAD, CRS therapy, contributing factors

## Abstract

**Background:**

Up to 40% of patients with chronic rhinosinusitis (CRS) remain uncontrolled despite guidelines of care being available, with an enormous socio-economic impact. The reasons for uncontrolled disease can be arbitrarily divided into disease-related, diagnosis-related, treatment-related, and patient-related factors. The relative contribution of each factor in uncontrolled CRS remains speculative. This explorative study aimed at determining the factors responsible for uncontrolled CRS in a tertiary care center, thereby identifying the most commons reasons for uncontrolled disease in CRS.

**Methods:**

Patients with uncontrolled CRS (*n* = 187) were asked to fill out a questionnaire and underwent a clinical examination at the outpatient clinic of the University Hospital of Leuven, Belgium. Two independent physicians evaluated the (multiple) reason(s) for uncontrolled disease.

**Results:**

In uncontrolled CRS, 66% of patients showed two or more reasons for uncontrolled disease according to the physicians' evaluation. Disease-related factors (70%) were most often considered the reason for uncontrolled disease, followed by treatment- (45%), patient- (42%), and diagnosis- (32%) related factors.

**Conclusion:**

In case of uncontrolled CRS, the different contributing factors to the uncontrolled nature need to be carefully addressed during diagnostic and therapeutic actions in order to define strategies to improve CRS care. Most uncontrolled CRS patients have multiple reasons contributing to their disease status, with disease-related factors being the most common factor.

## Introduction

Chronic rhinosinusitis (CRS) is an inflammatory condition of the nose and paranasal sinuses affecting up to 10,9% of the general population (1). Nasal endoscopy defines the major phenotypes, being CRS without (CRSsNP) and with nasal polyps (CRSwNP), while immune investigations reveal the endotypes (2, 3). Given the novel therapeutic options for CRSwNP, including biologicals, type 2 inflammation is getting progressively more associated with CRSwNP, as well as with comorbidities like asthma. However, underlying immune responses are not the only factor driving the multifactorial aetiology of CRS. Beside immune factors, also genetic, environmental, occupational, anatomic and iatrogenic factors contribute to the manifestation of CRS. In a single patient, it is often challenging to pinpoint one or multiple factors driving the chronicity of disease (4, 5). European and US guidelines can offer some guidance for symptomatic CRS care based on scientific evidence and level of control (6, 7). Nevertheless, not much is known about the different reasons underlaying uncontrolled CRS (8). Therefore, it is important to identify factors contributing to uncontrolled CRS allowing improvement of treatment strategies and patient care.

**Figure 1:**
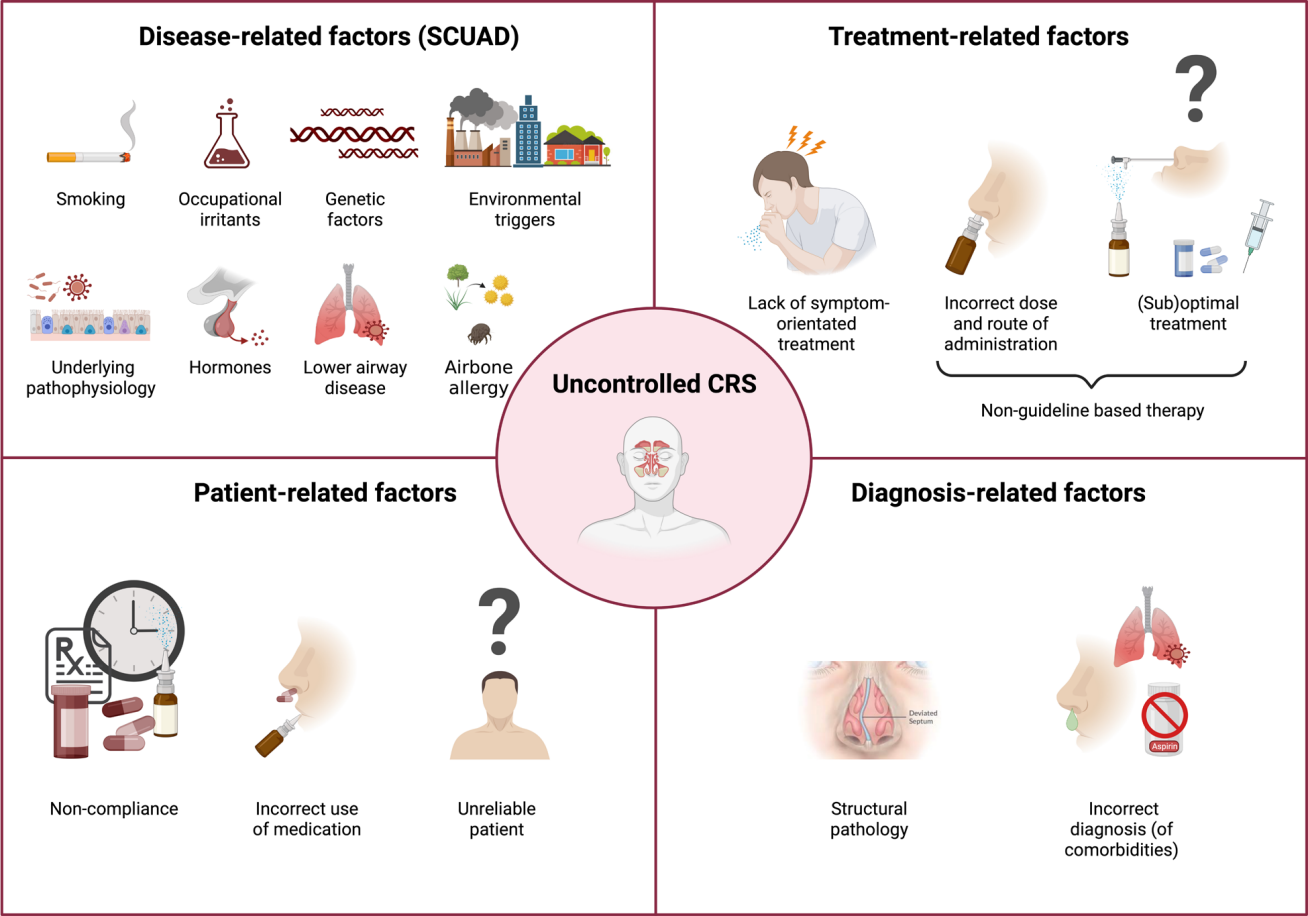
Reasons for uncontrolled chronic rhinosinusitis hellings and colleagues reviewed and classified the reasons for uncontrolled chronic rhinosinusitis (CRS) in the following four groups: disease-related factors, diagnosis-related factors, patient-related factors, and treatment-related factors (Hellings *et al*., 2013).

Disease control is defined as the disease state in which the patient subjectively does not experience symptoms anymore, or the remaining symptoms are not regarded as bothersome (4). More specifically, CRS patients are defined as controlled, partially controlled or uncontrolled according to the European Position Paper on Rhinosinusitis and Nasal Polyp (EPOS) criteria, where the degree of symptom reduction, the clinical mucosal aspect, the presence of adverse events, the need for systemic medication and the need for functional endoscopic sinus surgery objectively determine the disease control status (4). Despite multiple guidelines of care, it is estimated that up to 40% of patients with CRS continue to experience bothersome symptoms despite guideline-driven treatment, including functional endoscopic sinus surgery (5, 6, 9). A key reason for this lack of therapeutic success is our current limited understanding of the mechanisms underlaying uncontrolled CRS disease. Therefore, as stated above, it is important to identify all factors contributing to uncontrolled CRS. We have previously proposed to classify the reasons of uncontrolled CRS and AR into four major groups (Figure 1) (4): the disease-related, the treatment-related, the diagnosis-related and the patient-related factors.

**Disease-related factors** consist of factors that are contributing directly to the pathophysiology of CRS. This also includes environmental and life-style factors like occupational irritants, pollutants or cigarette smoke, which might contribute to the severity of CRS (10). Another example of a disease-related factor is a viral infection that predisposes some patients to recurrent bacterial rhinosinusitis episodes, thereby causing extensive damage to the ciliated cells and respiratory epithelium (e.g., Influenza, Adenovirus) (11, 12). Furthermore, also hormonal and genetic factors have been proposed to aggravate inflammation and the severity of upper airway diseases such as CRS (6). A real therapeutic challenge has been the severe chronic upper airway disease (SCUAD) patients as they are uncontrolled CRS patients with disease-related factors contributing to the severity of their disease. A better understanding of the prevalence and nature of all the contributing factors to their uncontrolled CRS state will therefore improve their treatment success (9, 11–13). **Treatment-related issues** of uncontrolled upper airway disease are linked with non-guideline based therapies consisting of (sub)optimal choice of treatment or an incorrect dose and route. Furthermore, also a lack of symptom-oriented treatment is categorized as a treatment-related factor. **Diagnosis-related factors** are factors that might be overlooked in an intial diagnosis that contribute to the uncontrolled disease state. For example, nasal hyperreactivity is a typical factor present in a subgroup of CRS patients, however it is often not diagnosed correctly (14). Other diagnosis-related factors include the presence of NSAID-exacerbated respiratory disease (N-ERD), structural nasal deformities, global airway dysfunction and systemic diseases or even an incorrect diagnosis of CRS. It is important to correctly diagnose these comorbidities as they have a significant impact on treatment strategy and patients need to be better informed about them. Lastly, **patient-related factors** consist of lack of compliance and treatment adherence, as well as the proper use of medication. A physician also needs to be attentive on how reliable the information given by the patient is.

The impact and prevalence of each of the four groups of factors responsible for uncontrolled disease will be evaluated in this study. The outcomes of this study will contribute to the improvement of treatment in uncontrolled CRS disease, as we will identify which factors contribute the most to uncontrolled disease. Better understanding of these factors will result in defined strategies to achieve disease control and increased quality of life (QoL) for patients, thereby reducing the socio-economic burden of uncontrolled CRS.

## Materials and methods

### Patient population

This academic observational trial was coordinated at the Department of Otorhinolaryngology, Head and Neck surgery of the University Hospital of Leuven, Belgium from 2016 until 2018, with analysis of data in 2021–2022. The study was evaluated and approved by the medical ethical commission of the university Hospital of Leuven (S57274). CRS patients were clinically examined by ENT specialists and asked to complete a questionnaire. The inclusion criteria were the following: an initial CRS diagnosis, a minimum of 17 years of age, a visual analogue scale (VAS) score of 5 or more for total sinus symptoms severity, and/or a SNOT-22 score of 35 or more despite previous or current medical/surgical treatment for their sino-nasal symptoms, and a minimum of two medical outpatient clinic visits for CRS in the last 5 years. Finally, to be eligible, the patient required at least 2 physician visits in the past for CRS to reduce the likelihood of including those without long-standing CRS or a false diagnosis of CRS. Exclusion criteria were being morbidly ill or recent sinus surgery (within the last 6 months).

A total of 200 uncontrolled CRS patients were screened of which 187 CRS patients met the inclusion criteria for evaluation of the reasons of uncontrolled CRS.

### Diagnosis of uncontrolled CRS

The initial diagnosis of uncontrolled CRS was assessed by ENT specialists (PVB and PH) at the University Hospital of Leuven based on CRS symptoms and clinical examination, including nasal endoscopy. All patients filled out a validated questionnaire which consisted of six parts:

• General information (age, gender, time to diagnosis of CRS, time to diagnosis, comorbidities, medication use, history of sinus surgery, and general health)
• Disease-related factors
• Diagnosis-related factors
• Treatment-related factors
• Patient-related factors
• The physician ‘s evaluation of the cause(s) for uncontrolled disease

The first five parts of the questionnaire were filled out by the patient. The last part of the questionnaire was completed by the physician after examination of the patient and then submitted to another physician who re-evaluated the reasons independently. Uncontrolled CRS was diagnosed using a VAS score of ≥5 and/or SNOT-22 score of ≥35 in combination with an evaluation of the nasal mucosa by nasal endoscopy (15).

The following symptoms on a VAS score were asked for further evaluation: total sino-nasal symptoms, nasal blockage, headache/facial pain, loss of smell, post-nasal drip (PND), runny nose, itchy eyes, itchy nose, sneezing, watery eyes, coughing, pressing sensation on the chest, dyspnoea, wheezing.

### Patient characteristics

General information included age, gender, onset of complaints, diagnosis of CRS, comorbidities, and general health. The time to diagnosis was determined by asking patients to describe the date of onset of the CRS symptoms and the date of the confirmation by means of clinical examination and nasal endoscopy. They were also asked to write down their comorbidities, which were verified using the patients' medical history in their medical record. Subjects were also asked to mark the state of general health in terms of excellent, good, moderate, or bad.

### Evaluation of disease-related factors

Several factors can be responsible for a severe phenotype of CRS. All patients were asked if their symptoms were triggered by occupational and/or environmental factors, which could contribute to the severity of the symptoms. Their medical history with a comorbidity of a lower-airway disease (COPD, chronic bronchitis, and asthma) and also the presence of a hypersensitivity to aspirin and other non-steroidal anti-inflammatory drugs (NSAIDs), was asked for. The patients were asked about nicotine abuse and the amount of smoking (packyears). Female sex hormones, which have been associated with more severe allergic inflammation, were evaluated by asking about oral contraceptives, pregnancy and menopause.

### Evaluation of diagnosis-related factors

The ENT specialist reconsidered the diagnosis of uncontrolled CRS and associated comorbidities, by investigating concomitant anatomic nasal deformities, global airway dysfunction, presence of N-ERD and systemic diseases, by medical history and clinical examination. Anatomic deformities included perforation of the septum, significant septal deviation and nasal valve dysfunction. In context of associated comorbidities, the presence of associated airborne allergies e.g., hay fever or house dust mite allergy was evaluated. Airway allergies were diagnosed by means of a skin prick test, Immunocaps/RAST or verified in the patient's medical record if test results within the last 2 years were available. Medical history of a systemic disease [congenital or acquired immunodeficiency, congenital mucociliary dysfunction, primary ciliary dyskinesia, cystic fibrosis, systemic vasculitis, or granulomatous diseases (e.g., granulomatosis with polyangiitis and sarcoidosis)], dental disease and gastroesophageal reflux were asked and checked in the medical files.

### Evaluation of treatment-related factors

Patients were asked about the current type of medication (e.g., nasal rinses, intranasal corticosteroids, antibiotics), dose, frequency, and route of administration were asked. The number of previous sinus operations was also questioned.

### Evaluation of patient-related factors

Patient-related factors including treatment adherence and correct medication use were questioned. Subjective factors like prejudices about treatment, fear of adverse events, and economic reasons were also asked. Patients were asked to report side effects. The doctor subjectively evaluated patient reliability.

### Statistics

Statistical analyses were performed with Graphpad Prism VI for Macintosh Version 8.4.3 (GraphPad Software Inc., San Diego, USA) by using the non-parametric Kruskal–Wallis test when comparing a single outcome between different groups. Prevalence differences in categorical variables (e.g., gender) were tested using the Pearson's chi squared test. A correlation analysis according to spearman was used to calculate a correlation between SNOT-22 score and the number of previous FESS operations. A statistical test was considered to be significant when *p* < 0.05.

## Results

### Patient characteristics and clinical characteristics

**Figure 2:**
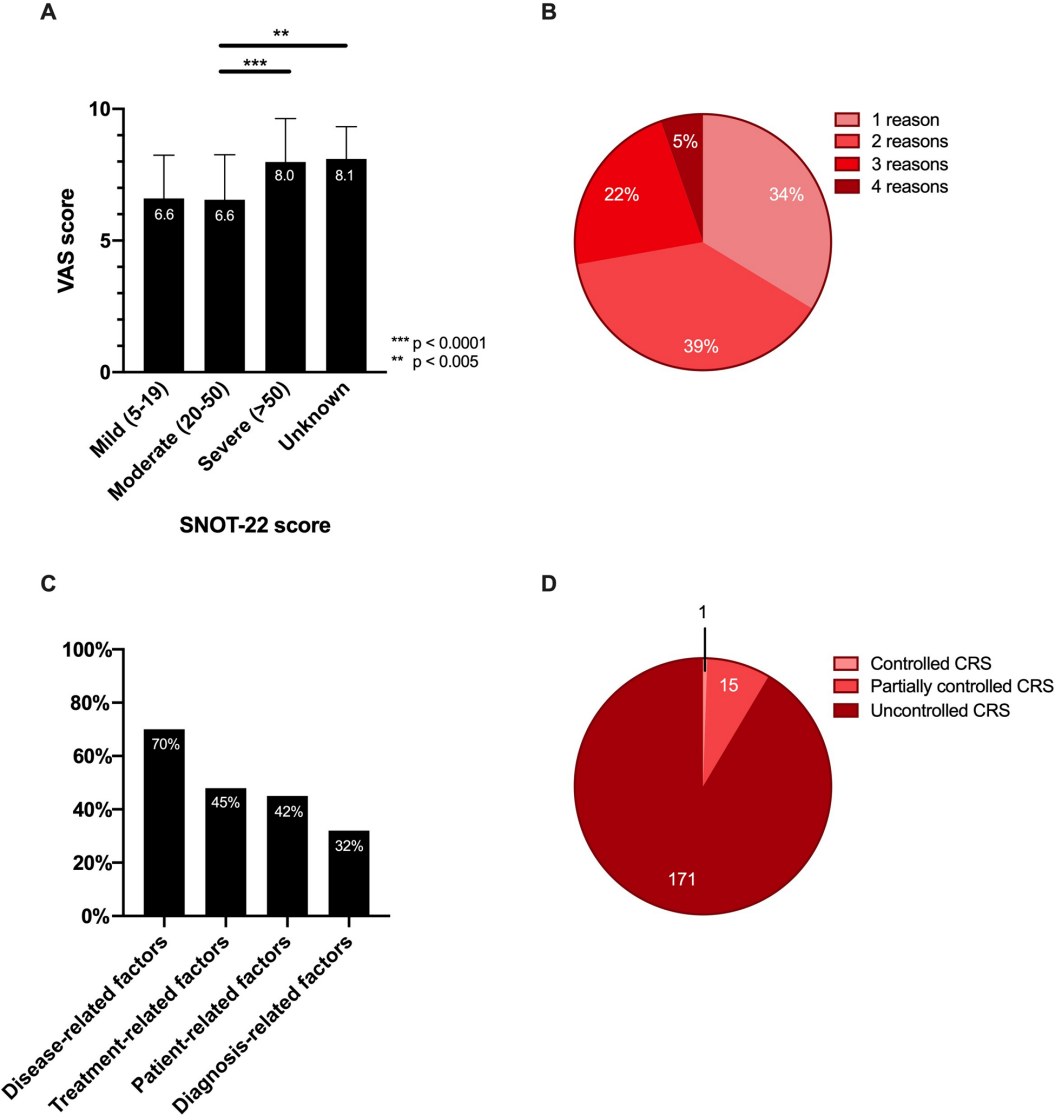
Average VAS score, number, and percentage of reasons for uncontrolled disease (**A**) average VAS score of patients in each SNOT-22 score group. Data are shown as mean ± standard deviation. Kruskal-Wallis test was used for comparison between the different groups; *** *p* < 0.0001; ** *p* < 0.007 (**B**) Number of reasons for uncontrolled disease as assessed by an ear-nose-throat (ENT) specialists based on the questionnaire and clinical evaluation. (**C**) Observed prevalence of each factor contributing to uncontrolled disease. (**D**) Disease status according to the EPOS 2020 guidelines for CRS in the patient cohort. We analyzed our inclusion criteria for uncontrolled CRS disease against the corresponding, more recently published, EPOS guidelines for uncontrolled disease in CRS patients (Fokkens *et al*., 2020). The numbers represent the number of patients falling into the corresponding EPOS guideline category for controlled, partially or uncontrolled CRS.

A total of 187 included patients with uncontrolled CRS were evaluated. The mean age was 46.56 ± 2.08 years with equal distribution between males (51%) and females (49%) (Table 1). Only 26% were diagnosed with CRS within the first year of their symptoms. More than half of the patients (62%) had airway comorbidities of which 32% had concomitant allergies and 25% had co-morbid asthma, while 44% had systemic comorbidities and 7% had N-ERD. The mean total VAS score of sino-nasal symptoms was high (7.39 ± 1.78) (Table 2). The complaints perceived as most bothersome were nasal obstruction, postnasal drip, headache and anosmia. The total VAS score of sino-nasal symptoms correlated well with the average SNOT-22 score. Patients with a high VAS-score also had a high SNOT-22 score (Figure 2A). In our cohort, most patients (83%) had 1 or 2 FESS operations in the past (Table 1). The number of FESS operations only had a small positive correlation with the SNOT-22 score (*p* < 0,01; r = 0,24). Most patients with CRS had two (39%) possible reasons for their uncontrolled disease (Figure 2B). A single cause was present in 34% of the patients, three reasons were present in 22% of the patients and only 5% had four reasons for uncontrolled CRS. The most frequent reason for uncontrolled CRS was a disease-related factor (70%) (Figure 2C). Treatment-related factors (45%) were reported on the second place, followed by patients-related factors (42%) and diagnosis-related factors (32%) (Figure 2C).

**Table 1:** General patient characteristics.

General information	
Age (years)	46,56 ± 2,08
Gender	
Male	51%
Female	49%
Time to diagnosis (years)	
<1	26%
1–5	27%
>5	20%
Unknown	27%
Number of previous FESS surgeries	
≤2	83%
>2	15%
>5	2%
Airway comorbidity	
Asthma	25%
COPD	2%
Chronic bronchitis	3%
Airway allergy	32%
Systemic comorbidity	44%
N-ERD	7%

General characteristics of the patient population. Abbreviations used COPD, chronic obstructive pulmonary disease; N-ERD, NSAID exacerbated respiratory disease; FESS, functional endoscopic sinus surgery. Age is shown as mean ± standard deviation.

**Table 2:** Mean visual analogue (VAS) score.

	VAS score ± SD
Total sino-nasal symptoms	7,39 ± 1,78
Nasal obstruction	6,50 ± 3,58
Postnasal drip	6,25 ± 2,85
Headache	6,10 ± 2,88
Anosmia/hyposmia	5,95 ± 3,26
Rhinorrhoea	4,90 ± 3,87
Sneezing	4,78 ± 2,82
Dyspnoea	3,85 ± 3,30
Epiphora	3,42 ± 2,87
Ocular itching	3,37 ± 3,08
Nasal itching	3,31 ± 2,73

Mean visual analogues (VAS) scores and their standard deviation of all the patients (*n* = 187). Data are shown as mean ± standard deviation. VAS scores were evaluated using questionnaires filled out by the patients.

### Disease-related factors

Concerning the disease-related factors, 61% of the patients had an environmental factor, such as exposure to cold air, fog, a hobby (e.g., swimming or painting) or contact with their pet, contributing to their symptoms. Occupational factors were reported in 35% of the patients of which the most common occupations were office workers or teacher/day care centre operator. Lower airway disease was seen in a 30% of the patients of which the majority had asthma (25%) contributing to their uncontrolled CRS, while COPD and chronic bronchitis contributed in 2% and 3% of the cases, respectively. Furthermore, airborne allergies were present in 32% of our patient cohort. Other disease-related factors contributing to uncontrolled disease were genetic factors in 5% of the patients and smoking in 20% of the patients. Hormones and pregnancy were only relevant in one subject. (Table 3A).

**Table 3:** Reasons for uncontrolled disease.

A: Disease-related factors	
Environmental triggers	61%
Occupational factors	35%
Lower airway disease	30%
Smoking	20%
Genetic factors	5%
Airborne allergies	32%
B: Treatment-related factors	
Non-guideline-based therapy	44%
Non-symptom oriented	11%
C: Patient-related factors	
Non-compliance	30%
Incorrect use of medication	29%
Unreliable	6%
D: Diagnosis-related factors	
Structural pathology	21%
Incorrect diagnosis (of comorbidities)	4%

Descriptive observation of the different subcategories contributing to uncontrolled disease in CRS patients categorised into the 4 main reasons for uncontrolled disease (Hellings *et al*., 2013).

### Treatment-related factors

A total of 44% patients had non-guideline recommended therapy according to the EPOS guidelines (6). Of these patients 20% received maintenance treatment but not the required medication according to the EPOS guidelines and 24% didn't even receive any maintenance therapy at all and relied solely on rescue medication. Of all patients in the cohort, 59% received rescue medication at least one time within the last 3 months (Table 4). Only 11% of patients had a lack of symptom-oriented treatment. (Table 3B).

**Table 4:** Relevant medication use of the patients.

Maintenance treatment	76%
Guideline treatment	56%
Non-guideline recommended treatment	20%
No treatment	24%
Rescue medication within the last 3 months	59%
Systemic corticosteroids	35%
Antibiotics	33%
Systemic corticosteroids & antibiotics	33%

Patients were asked about their current maintenance treatment. The physicians determined whether the treatment was sufficient according to the EPOS guidelines for chronic rhinosinusitis (Fokkens *et al*, 2020). Rescue medication, consisting of systemic corticosteroids, antibiotics, or both, used within the last 3 months were questioned in the patient cohort.

### Patient-related factors

When looking at the patient-related factors, 30% of the patients suffered from uncontrolled disease due to poor treatment adherence. Furthermore, 29% used their medication incorrectly, while 6% of the patients were deemed unreliable by their physicians. Side effects of treatment occurred in 12% of patients. The most frequent side effects reported were sleepiness and epistaxis. Approximately 19% of the patients stopped their medical treatment for a certain amount of time, because they were tired of taking medication every day or didn't deem it necessary anymore (Table 3C).

### Diagnosis-related factors

In 21% of the patients, structural nasal pathology was identified as a diagnosis-related factor contributing to uncontrolled disease. Significant nasal septal deviation was seen in 11% of patients, nasal valve dysfunction in 6% and septal perforation in 3%. The initial diagnosis of CRS was incorrect in 4% of the patients who mostly had rhinitis medicamentosa or postnasal drip due to allergic or non-allergic rhinitis. (Table 3D).

### Correlation of the definitions for uncontrolled disease

During the course of this study, new EPOS guidelines (6) were published on defining (un)controlled CRS. We correlated our population of uncontrolled CRS patients with the new guidelines and saw that, as expected, more than 90% of these patients were also defined as uncontrolled according to the new EPOS guidelines (Figure 2D). Out of the 187 patients in our cohort, fifteen patients were defined as partially controlled, and one patient was classified as controlled according to the new EPOS guidelines. The controlled status according to the new EPOS guidelines of this one patient can be attributed to a low VAS score for specific CRS symptoms such as postnasal drip despite a high overall VAS score for total sino-nasal symptoms.

## Discussion

This study found that disease-related factors (SCUAD) were the most common factors underlaying uncontrolled disease in CRS patients followed by treatment-, patient-, and diagnosis- related factors. Most patients reported two or more reasons for poor CRS control. In addition, this study found that only 26% of patients were correctly diagnosed with CRS within one year after the start of their symptoms.

SCUAD was found in an astonishing 70% of the uncontrolled CRS population. These SCUAD patients represent a real therapeutic challenge for physicians because these disease-related factors are hard to modify or treat. For example, the environmental and occupational factors, which were the most common disease-related factors, cannot be that easily modified as other factors. Nevertheless, most of these SCUAD patients also had other factors contributing to their uncontrolled disease of which patient-related factors were the most common second factor. Other factors, and especially these patient-related factors, can be more easily managed compared to the disease-related factors (16). Therefore, it might be a therapeutic strategy to eliminate other factors as much as possible in these SCUAD patients, which can lead to better disease control.

Treatment-related factors are the second most common reason of uncontrolled CRS. Twenty-four percent of these patients didn't receive any maintenance treatment despite being recommended by the EPOS guidelines. Non-compliance was the most common reason of the patient-related factors, which is a known phenomenon in chronic diseases (17). Since 29% of patients did not use their medication correctly, there is a need to better educate the patients how to use their medication e.g., point the nozzle of the intranasal corticosteroid slightly outwards, away from the septum while spraying (18). Recent technologies, such as mobile applications, pave the way for improving patient-related factors. For example, mobile applications, such as “mySinusitisCoach” (19) can be specifically designed for better follow up of the patient's compliance and treatment adherence. Additionally, a mobile application can also contain tips and tricks, as well as videos on how to correctly use medication, therefore better educate the patient in how to use their medication (19, 20).

As expected, due to our inclusion criteria this study confirmed a high average VAS-score (7.39 ± 1.78) for total sino-nasal symptoms in uncontrolled CRS patients. The study also confirmed the classic symptoms of CRS (nasal obstruction, postnasal drip, headache and anosmia). A remarkable finding is that only 26% of patients were correctly diagnosed with CRS within one year after the start of their symptoms. However, confirmation of the above percentage with other studies in the future is necessary. In our cohort, 20% of the patients were smokers which is slightly higher than the national average of 12% (21). The number of patients who had concomitant asthma and/or airborne allergies were in line with current literature (22).

Current literature often describes the epidemiology of CRS and how to classify severe uncontrolled CRS patients (6, 8, 23). However, there is a lack of studies covering the actual reasons behind patients being uncontrolled after years and years of treatment (24). Therefore, our study, which defines the different factors important for uncontrolled disease, paves the way for the optimalisation of CRS care. As still up to 40% of CRS patients remain uncontrolled despite guideline-based treatments, a better characterisation of these four main factors in uncontrolled CRS patients will help to set up a patient-centred treatment plan, specifically managing an individual patients' needs.

One of the strengths of this study is the large number of patients despite being a monocentric study. Another strength is the appropriate clinical and anamnestic parameters to define uncontrolled disease. We defined CRS as uncontrolled if the VAS-score for total sino-nasal symptoms was 5 or more and/or if the SNOT-22 score was 35 or more. The former because a VAS score of 5 or more has been generally accepted in the field to significantly affect the QoL of the patients and has more recently also been incorporated in the latest EPOS guidelines and EUFOREA guidelines for severe uncontrolled CRS (6, 23). The latter because a SNOT-22 score of 35 or more was identified as the best cut-off for poorly controlled severe CRS patients (25). This SNOT-22 cut-off of 35 or more was also later confirmed by the recent EPOS and EUFOREA guidelines for severely uncontrolled CRS (6, 23). During the course of our study new EPOS guidelines (6) were published on defining (un)controlled CRS. Our correlation analysis showed that more than 90% of the patients in our cohort were still defined as having “uncontrolled CRS” according to the new guidelines. There was only one patient that, due to a low VAS-score for specific CRS symptoms, despite having a high overall VAS score for total sino-nasal symptoms, was deemed controlled according to the new criteria EPOS criteria (6).

Our study also has limitations. Although the VAS-score and SNOT-22 score were combined with an endoscopic evaluation of the nasal mucosa, we cannot deny that all these tests are rather subjective, and endoscopy is susceptible to interobserver variability. It would be more objective if a low-dose CT-scan was performed in all patients (26). In contrast, a strength of this study is that airborne allergies were objectified using a skin or RAST test. However, we did not discriminate between CRSwNP and CRSsNP, it could be interesting for future research to discriminate between these two phenotypes as this might reveal different contributing factors based on phenotype.

To conclude, the most common reason for uncontrolled CRS is related to the disease itself, followed by treatment-, patient-, and diagnostic related factors. However, most patients reported two or more reasons for poor CRS control. Therefore, it is important to properly identify the underlying reasons for uncontrolled disease in CRS patients and focus on easily manageable contributing factors to optimise CRS care.

## Data Availability

The raw data supporting the conclusions of this article will be made available by the authors, without undue reservation.
